# Integrative Functional Genomic Analysis of Molecular Signatures and Mechanistic Pathways in the Cell Cycle Underlying Alzheimer's Disease

**DOI:** 10.1155/2021/5552623

**Published:** 2021-07-11

**Authors:** Zhike Zhou, Jun Bai, Shanshan Zhong, Rongwei Zhang, Kexin Kang, Xiaoqian Zhang, Ying Xu, Chuansheng Zhao, Mei Zhao

**Affiliations:** ^1^Department of Geriatrics, The First Affiliated Hospital, China Medical University, Shenyang, 110001 Liaoning, China; ^2^Cancer Systems Biology Center, The China-Japan Union Hospital, Jilin University, Changchun, 130033 Jilin, China; ^3^Department of Neurology, The First Affiliated Hospital, China Medical University, Shenyang, 110001 Liaoning, China; ^4^Computational Systems Biology Lab, Department of Biochemistry and Molecular Biology and Institute of Bioinformatics, University of Georgia, USA; ^5^Department of Cardiology, The Shengjing Affiliated Hospital, China Medical University, Shenyang, 110004 Liaoning, China

## Abstract

**Objective:**

Alzheimer's disease (AD) is associated with cell cycle reentry of mature neurons that subsequently undergo degeneration. This study is aimed to identify key regulators of the cell cycle and their underlying pathways for developing optimal treatment of AD.

**Methods:**

RNA sequencing data were profiled to screen for differentially expressed genes in the cell cycle. Correlation of created modules with AD phenotype was computed by weight gene correlation network analysis (WGCNA). Signature genes for trophic factor receptors were determined using Pearson correlation coefficient (PCC) analysis.

**Results:**

Among the 13,679 background genes, 775 cell cycle genes and 77 trophic factor receptors were differentially expressed in AD versus nondementia controls. Four coexpression modules were constructed by WGCNA, among which the turquoise module had the strongest correlation with AD. According to PCC analysis, 10 signature trophic receptors most strongly interacting with cell cycle genes were filtered and subsequently displayed in the global regulatory network. Further cross-talking pathways of signature receptors, such as glutamatergic synapse, long-term potentiation, PI3K-Akt, and MAPK signaling pathways, were identified.

**Conclusions:**

Our findings highlighted the mechanistic pathways of signature trophic receptors in cell cycle perturbation underlying AD pathogenesis, thereby providing new molecular targets for therapeutic intervention in AD.

## 1. Introduction

Alzheimer's disease (AD) accounts for 60-70% of all dementia in the elderly and thus imposes a heavy burden on society [1, 2]. This clinical entity is a slowly progressing brain disorder manifested by cognitive decline, behavioral abnormality, and psychiatric alteration [3]. Pathologically, the core hallmarks of AD are neurofibrillary tangles composed of hyperphosphorylated tau and senile plaques consisting of beta-amyloid (A*β*) peptides [4]; besides, synaptic and neuronal deficits are also characteristic of the disease [5, 6]. Neuron demise in AD has been proposed to be attributable, at least in part, to abnormal activation of cell cycle proteins and DNA tetraploidy followed by neuronal reentry into the cell cycle [2]. Indeed, the cell cycle is a tightly regulated process that, when reinitiated or undergoing failure of cell cycle arrest, may result in a pattern of programmed cell death termed as apoptosis [2, 7, 8]. This unscheduled event in turn facilitates A*β* toxicity and tau hyperphosphorylation, thus to the formation of AD neuropathology [9, 10].

Progress in the cell cycle depends on the coordination and interaction of trophic factors with two categories of regulatory proteins, including cyclins and cyclin-dependent kinases (CDKs) [11, 12]. For instance, nerve growth factor (NGF) inhibits the induction of cyclins and their associations with specific CDKs, hence interrupting cell cycle reentry through the G1-phase [13]. Insulin-like growth factor 1 (IGF-1) prevents the expression of cell cycle proteins (e.g., cyclin A, D1, and CDK2) known to stimulate G1 quiescent cells to enter the S-phase [14]. Notably, both NGF and IGF-1 have been found to be downregulated in AD, with the extent of downregulation proportional to A*β* burden and poor cognition [15, 16]. Additional evidence also supports the regulatory role of trophic factors in the cell cycle, such as epidermal growth factor [17], endothelial growth factor [18], and fibroblast growth factor [19]. Intriguingly, the expression of corresponding receptors for these factors presents periodic variations throughout the cell cycle, giving rise to intermittent effects of the ligands on neurons at different phases of the cell cycle [20].

Based on such observations, we preliminarily infer that neuronal death following cell-cycling initiation is implicated in AD pathogenesis, at least partially due to insufficient support from trophic factors (either downregulation or intermittent effect). This is of profound clinical significance, as understanding AD neurodegeneration mediated by early trophic receptor processing and signaling events in the cell cycle offers promise for those who seek therapeutic interventions, which may contribute to slowing or even blocking the occurrence and progression of AD. Accordingly, we collected cell cycle-encoding genes as well as trophic factor receptors in incident AD dementia by reviewing existing literature. Subsequently, an integrative genomic analysis was performed on basis of gene expression profiles and functional annotations, aiming to (1) provide a computational validation for the involvement of cell cycle in AD onset, (2) identify key regulators as attractive therapeutic targets in a cell cycle subnetwork, and (3) elucidate the underappreciated therapeutic targets in the pathogenesis of AD.

## 2. Materials and Methods

### 2.1. Data Resources

All RNA sequencing (RNA-seq) and microarray data of temporal cortex tissues from AD patients and nondementia controls were downloaded through Gene Expression Omnibus (GEO, https://www.ncbi.nlm.nih.gov/geo/) database [21].  Table 1 exhibited the information of selected datasets and the number of samples for analysis. Sex-matching was observed between 176 AD cases (male/female: 89/87) and 160 nondementia controls (male/female: 85/75; *p* = 0.64). The mean age was 83.60 ± 8.25 years (range: 40-105 years) for AD and 81.38 ± 10.78 years (range: 43-102) for nondementia. Clinical phenotypic data of samples are detailed in Supplementary Table S1. Subjects were clinically and/or pathologically diagnosed with AD had to be based on certain standardized criteria, e.g., the National Institute of Neurological and Communicative Disorders and Stroke-Alzheimer's Disease and Related Disorders Association (NINCDS-ADRDA) [22]; the International Classification of Diseases- (ICD-) 10 criteria, and the Diagnostic and Statistical Manual of Mental Disorders- (DSM-) III, -IV, or -V criteria [23–26]; the Consortium to Establish a Registry for Alzheimer's Disease (CERAD) guidelines [27]; and the Braak stage [4]. The list of cell cycle genes was obtained from a published study (see Supplementary Table S2) [28]. A total of 169 receptors for cell cycle-related trophic factors was collected based on our literature review (see Supplementary Table S3).

### 2.2. Gene Set Enrichment Analysis (GSEA)

Gene expression profiles from five datasets (GSE132903, GSE118553, GSE5281, GSE37264, and GSE36980) were merged into a new dataset. The *limma* package of R software was used to eliminate batch effects during the merging process. As shown in Table 2, biological processes (BP) of gene ontology (GO) terms significantly enriched in AD phenotype were filtered by GSEA [29, 30]. The number of permutations was set to 1000, and *p* < 0.05 was considered statistically significant. The visualization of GSEA was accomplished using *ClusterProfler*, *enrichplot*, *ggplot2*, and *GSEABase* packages.

### 2.3. Identification of Differentially Expressed Genes (DEGs)

Differential expression analysis of genes was conducted by comparing RNA-seq data between AD and control tissues adopting *lmFit* and *eBayes* functions. Analyses of two-dimensional hierarchical clustering and volcano plot for DEGs were performed using *limma*. A false discovery rate- (FDR-) adjusted *p* < 0.05 and fold change (FC) ≥ 1.2 were defined as differentially expressed [31–33].

### 2.4. Coexpression Network Analysis and Signature Trophic Receptors

The cell cycle genes (Supplementary Table S2) and trophic receptors (Supplementary Table S3) obtained from our literature review were merged to match the DEGs. The overlapping genes (defined as cell cycle-related genes) and their coexpression modules were identified by weighted correlation network analysis (WGCNA) [34], with the differential expression of genes given in Supplementary Table S4. During clustering analyses, the genes that were heavily involved in noncell cycle processes were grouped into the grey module, as their expression levels might not necessarily represent the actual level of cell cycle activity in AD. The correlation of cell cycle genes with trophic receptors was computed by Pearson correlation coefficient (PCC). The association of cell cycle-related genes with their phenotypes (i.e., AD, age, and gender) was measured by gene significance (GS). Under the premise of p.GS <0.05 in AD phenotype, the top 10 receptors were identified as the signature trophic receptors, the expression of which had the strongest correlation with cell cycle genes (Table 3). Detailed data of PCC and GS for each receptor are shown in Supplementary Table S5. Functional enrichment analyses of Kyoto Encyclopedia of Genes and Genomes (KEGG) pathways were carried out using *clusterProfiler*.

### 2.5. Global Regulatory Network and Cross-Talking Pathways of Signature Trophic Receptors

Based on the STRING database (Search Tool for the Retrieval of Interacting Genes, https://www.string-db.org/) [35], the module with the strongest correlation with AD was selected to construct the global regulatory network. Thereafter, *cytoscape* software was utilized to visualize the global regulatory network and cross-talking pathways of signature trophic receptors [36].

### 2.6. Analysis of Area under the Curve (AUC)

The *pROC* function was adopted to estimate the diagnostic performance of signature trophic receptors in differentiating AD from controls. Generally, a complete prediction was indicated by an AUC value of 100%, whereas a random selection was represented by 50%. All *p* values were bilateral with *p* < 0.05 considered of statistical significance.

## 3. Results

### 3.1. Differentially Expressed Genes and GESA of Biological Processes

**Figure 1:**
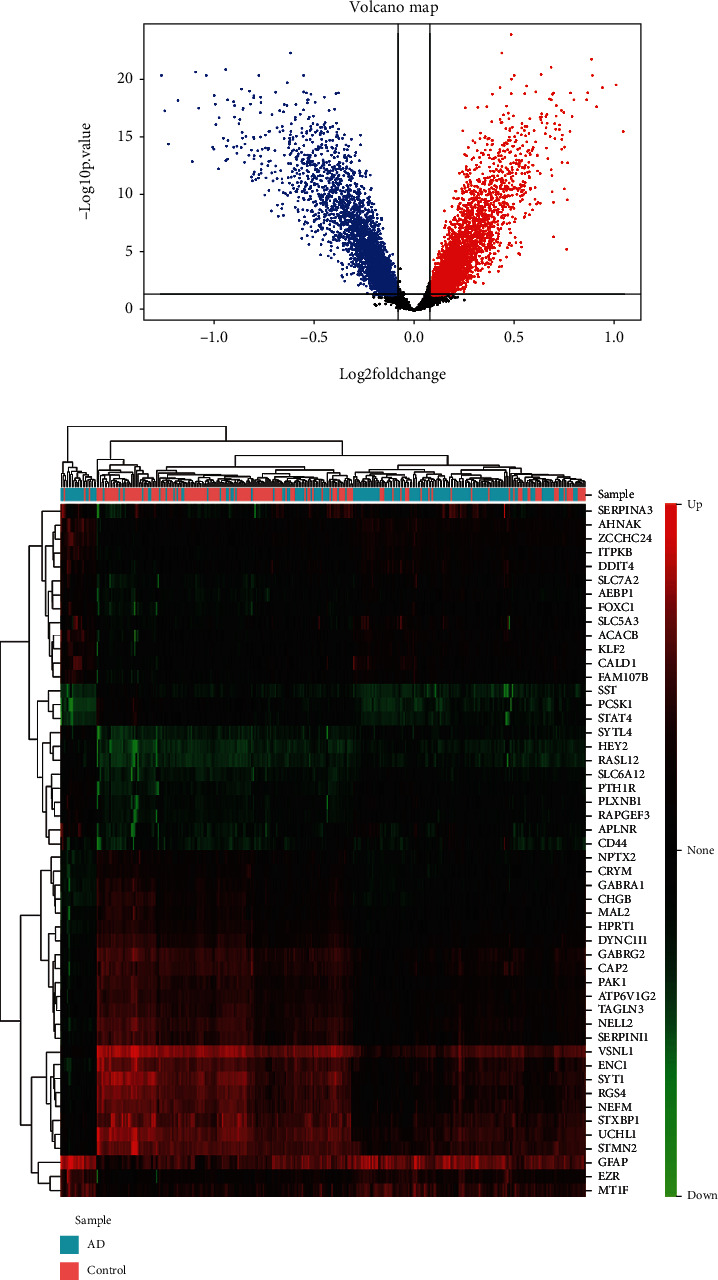
Differential expression analysis. (a) Volcano plot of DEGs between AD and nondementia controls. (b) Heatmap of the top 25 down- and upregulated DEGs: green to red indicates the process from down- to upregulation of gene expression. AD: Alzheimer's disease; DEGs: differentially expressed genes.

After removing unannotated and duplicate genes, 6,847 out of 13,679 background genes were differentially expressed between AD and nondementia controls (see Methods) (Figure 1(a)). There were 852 cell cycle-related genes (including 775 cell cycle genes and 77 trophic receptors) that overlapped with these DEGs (Supplementary Table S4). Heatmap of DEGs with the top 25 up- and downregulated expression in AD and nondementia controls was exhibited in Figure 1(b). In AD, the major enrichment of BP (Table 2) was involved in the regulation of cell proliferation, programmed cell death, and apoptotic processes, as well as negative regulation of nucleic acid-templated transcription, RNA biosynthetic, and metabolic processes.

### 3.2. Coexpression Modules and Functional Enrichment Analysis

**Figure 2:**
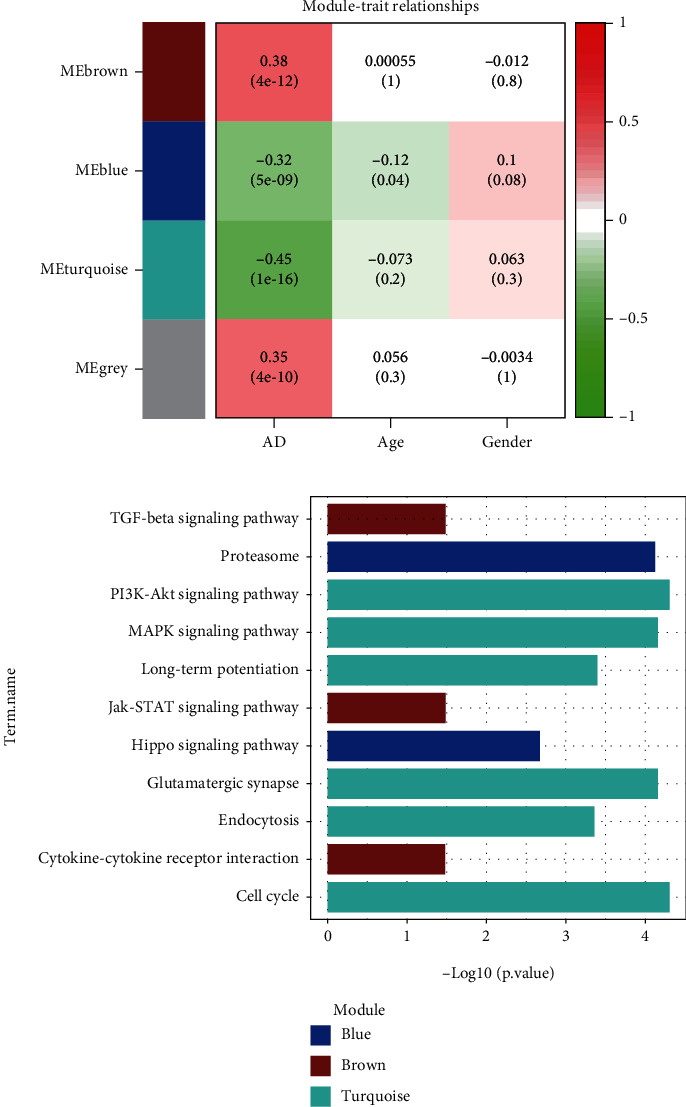
Weighted correlation network analysis. (a) Module-trait relationships of created modules: green to red represents the correlation of modules from negative to positive with phenotypes. (b) Enrichment of KEGG pathways on functional modules. AD: Alzheimer's disease; KEGG: Kyoto Encyclopedia of Genes and Genomes.

Three hundred and eight samples passed the cut-off line with a height of 25, which were hierarchically clustered by the average linkage. Four created modules were established by WGGNA, among which the grey module was composed of noncoexpressed genes, indicating them to be involved in noncell cycle processes. Heatmap of module-trait relationships (Figure 2(a)) presented the most significant negative correlation of turquoise module (correlation coefficient = −0.45, *p* = 1*e* − 16), as well as a positive correlation of blue (correlation coefficient = 0.32, *p* = 5*e* − 09) and brown (correlation coefficient = 0.38, *p* = 4*e* − 12) modules with AD phenotype. Annotation of KEGG pathway (Figure 2(b)) was performed by functional enrichment analysis, which revealed that the DEGs in the blue module were involved in proteasome and Hippo signaling pathways; the DGEs of brown module participated in cytokine-cytokine receptor interaction, TGF-beta, and Jak-STAT signaling pathways; the DEGs in the turquoise module were enriched in cell cycle, endocytosis, glutamatergic synapse, long-term potentiation, PI3K-Akt, and MAPK signaling pathways.

### 3.3. Module-Pathway Regulatory Network and AUC Analysis

**Figure 3:**
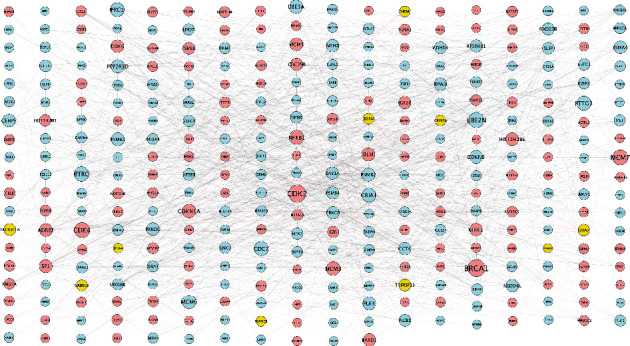
Global regulatory network. Global regulatory network of cell cycle-related genes: yellow represents the signature trophic receptors; blue indicates low expression; red represents high expression; node size reflects the degree of gene connectivity.

**Figure 4:**
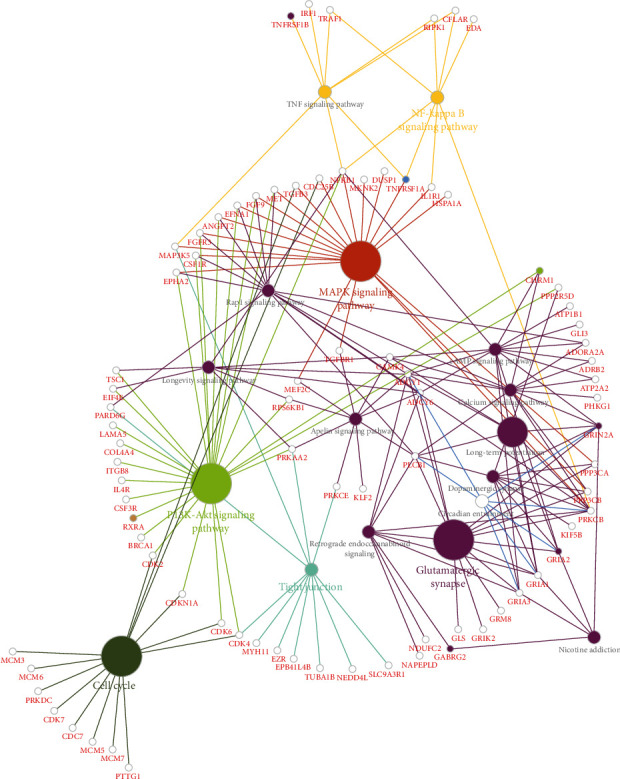
Cross-talking pathways. Cross-talking pathways of signature trophic receptors: small colored nodes indicate the signature trophic receptors.

**Figure 5:**
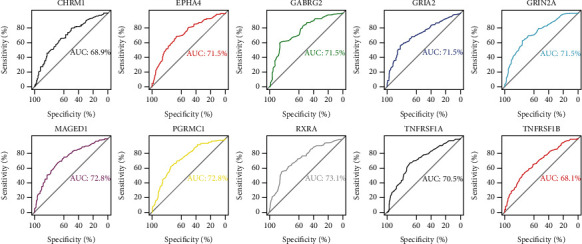
AUC analyses of trophic receptors. AUC: area under the curve.

Ten signature receptors (GRIN2A, GRIA2, CHRM1, GABRG2, PGRMC1, EPHA4, MAGED1, TNFRSF1B, TNFRSF1A, and RXRA) interacting with cell cycle genes were displayed in the global regulation network (Figure 3), thus to enrich the cross-talking pathways of signature receptors in AD. As shown in Figure 4, GRIN2A (i.e., glutamate ionotropic receptor NMDA type subunit 2A) belonging to a family of glutamate-gated ion channel receptors, participated in the glutamatergic synapse, long-term potential, cAMP, and calcium signaling pathways. GRIA2 (i.e., glutamate ionotropic receptor AMPA type subunit 2) encodes a member of the glutamate receptor family that is activated in numerous neuropathological processes [37–39]. This gene was involved in the dopaminergic synapse, long-term potential, and calcium signaling pathway. CHRM1 (i.e., cholinergic receptor muscarinic 1) belongs to a muscarinic cholinergic receptor in the larger family of G protein-coupled receptors, which was enriched in PI3K-Akt, cAMP, and calcium signaling pathways. GABRG2 (i.e., gamma-aminobutyric acid type A receptor subunit gamma2) encodes a receptor of gamma-aminobutyric acid (GABA) that is the major inhibitory neurotransmitter in the mammalian brain [40]. This gene participated in retrograde endocannabinoid signaling and nicotine addiction. RXRA (i.e., retinoid X receptor alpha) is a nuclear receptor mediating the biological effects of retinoids by participating in retinoic acid-mediated gene activation. The functional enrichment analysis showed this gene enriched in the PI3K-Akt signaling pathway. TNFRSF1A (i.e., TNF receptor superfamily member 1A), belonging to a member of the TNF receptor superfamily, was involved in MAPK, TNF, and NF-Kappa B signaling pathways. TNFRSF1B (i.e., TNF receptor superfamily member 1B) encoding another member of the TNF-receptor superfamily of proteins, participated in TNF signaling pathway. In brief, the signature receptors are mainly involved in the glutamatergic synapse (GRIN2A), long-term potential (GRIN2A, GRIA2), MAPK (TNFRSF1A), and PI3K-Akt (CHRM1, RXRA) signaling pathways (Figure 4). Analysis of AUC exhibited a good diagnostic performance of each signature receptor in predicting AD onset (Figure 5).

## 4. Discussion

To examine the relevance of the cell cycle in the AD brain, we took advantage of publicly available RNA-Seq data from 336 postmortem human samples to identify 775 cell cycle genes and 77 trophic receptors that were differentially expressed between AD and nondementia controls. The reason for choosing temporal lobe tissues for our investigation was on basis of the region's high susceptibility to neuron loss during neurodegeneration of AD [41]. Analytic results of GSEA showed that DEGs of the cell cycle were enriched in regulation of cell proliferation, programmed cell death, and apoptotic processes, as well as negative regulation of nucleic acid-templated transcription, RNA biosynthetic, and metabolic processes. Of note, these biological processes apparently pointed towards a confrontational interaction between cell demise and proliferation, supporting the underlying manifestation of cell cycle dysfunction in the human AD brain.

The results emerging from WGCNA revealed that brown, blue, and turquoise modules were intimately associated with AD, the DEGs of which were enriched in proteasome, cytokine-cytokine receptor interaction, glutamatergic synapse, long-term potentiation, endocytosis, PI3K-Akt, and MAPK signaling pathways. Accumulating evidence suggests that perturbations in the glutamatergic synapse, consisting of the presynaptic terminal, astrocytic process, and postsynaptic spine, underlie the pathogenic mechanisms of AD [42–44]. As the primary excitatory neurotransmitter, glutamate has been found to be implicated in hippocampus-dependent learning and memory functions [45, 46]. A*β* induces extracellular glutamate release to provoke overexcitation of N-methyl-D-aspartate receptors (NMDARs), which is an excitotoxic process relevant to neuronal degeneration, cell damage, and apoptosis [47, 48]. Alternatively, this excitatory toxicity might also occur at the normal level of glutamate due to overstimulation of glutamate receptors, such as tau-induced alterations in NMDARs phosphorylation [49]. There were several pieces of evidence that glutamate bound to NMDARs to undergo a conformational change and thus to open ion channels [50–52]. The resultant influx of calcium initiated secondary messenger systems, giving rise to the induction of long-term potentiation, an activation process for synaptic plasticity and memory processing in hippocampus [53]. Evidence shows that either A*β*-induced glutamate release [54] or overactivation of NMDARs [33] has dramatic consequences for an overload of intracellular calcium, leading to impaired long-term potential, hyperphosphorylation of tau, and synaptic loss [48, 55].

In terms of MAPK and PI3K-Akt signaling, neurotrophin receptors couple with these pathways are responsible for many critical processes in AD, as confirmed ranging from synaptic plasticity to neuronal growth and apoptosis [56]. The MAPK signaling constitutes a primary conduit to the genomic response of synaptically activated neurons essential for long-term recognition memory [57, 58]. Specifically, oxidative stress induced by A*β* affects MAPK cascade activity, which inhibits astrocytic uptake of glutamate and upregulated NMDARs, resulting in cognitive impairment and AD neurodegeneration [59]. In addition, PI3K signaling was found to participate in the homeostatic regulation of apoptosis and memory consolidation by inhibiting proapoptotic transcription factors [60, 61]. In the case of GSK3*β* factor, for instance, it is a ubiquitously expressed serine/threonine kinase interacting with extrasynaptic NMDARs in PI3K-Akt signaling, which contributes to tau phosphorylation, A*β* generation, and neuronal death, hence implying the involvement of trophic receptors in AD pathogenesis through the PI3K-Akt signaling pathway [62, 63].

We identified 10 signature trophic receptors (GRIN2A, GRIA2, CHRM1, GABRG2, PGRMC1, EPHA4, MAGED1, TNFRSF1B, TNFRSF1A, and RXRA) suggestive of a robust association with AD. The strong molecular and disease phenotypes from the created modules in our study determined these receptors as key regulators of AD pathogenesis, and that their mechanistic pathways were considered promising candidates for the treatment of AD. Some signature trophic receptors have already been approved or tested as drug targets for AD. GRIN2A encodes the GluN2A subunit of the NMDAR, which, according to its neuroexcitatory toxicity, is responsible for synaptic loss [64–66]. In view of this property, memantine, an NMDAR antagonist, has been developed for treating moderate-to-severe AD [67]. Deficient RNA editing of the GRIA2 Q/R site has been found to have pathogenic effects, which could be a target of novel therapeutic strategies in AD [68]. CHRM1 is a muscarinic cholinergic receptor that is considered another crucial antidementia target. In practice, acetylcholinesterase inhibitors (e.g., donepezil, rivastigmine, galantamine, and tacrine) have been widely used in the clinical treatment of mild-to-moderate AD [69, 70]. Remarkably, all these signature trophic receptors exerted a good diagnostic performance in predicting AD according to AUC analysis, supporting them to be potential biomarkers of AD. Further *in vivo* or *in vitro* experiments are expected to validate the relevant pathways of the cell cycle underlying the pathological process of AD.

## 5. Conclusions

In aggregate, gene expression profiling is a promising approach to reveal the intricate mechanisms of the cell cycle underlying AD. Dysregulation of key trophic factor receptors in the cell cycle is involved in the pathogenesis of AD, possibly mediated via glutamatergic synapse (GRIN2A), long-term potential (GRIN2A, GRIA2), MAPK (TNFRSF1A), and PI3K-Akt (CHRM1, RXRA) signaling pathways. Our findings illuminate the role of the cell cycle in AD neurodegeneration, which may fuel interest in the interaction of cell cycle regulation with AD therapy by targeting signature trophic receptors.

## Figures and Tables

**Table 1:** The number of samples for both AD and non-dementia controls in included datasets.

Tissue	GEO	Platform	AD	Controls
Temporal lobe	GSE132903	GLP10558	97	98
	GSE118553	GPL10558	45	24
	GSE5281	GPL570	16	11
	GSE37264	GPL5188	8	8
	GSE36980	GPL6244	10	19

AD: Alzheimer's disease; GEO: Gene Expression Omnibus.

**Table 2:** Biological processes of gene set enrichment analysis.

ID	Description	Enrichment score	*p* value
GO:0007267	Cell-cell signaling	-0.339309387	0.002159827
GO:0051649	Establishment of localization in cell	-0.333291297	0.002183406
GO:0042127	Regulation of cell proliferation	0.329444498	0.001858736
GO:1902679	Negative regulation of RNA biosynthetic process	0.328198078	0.001992032
GO:1903507	Negative regulation of nucleic acid-templated transcription	0.328198078	0.001992032
GO:0003008	System process	-0.32530293	0.001996008
GO:0043067	Regulation of programmed cell death	0.321860897	0.001941748
GO:0055085	Transmembrane transport	-0.319180234	0.00204918
GO:0051253	Negative regulation of RNA metabolic process	0.31829589	0.001945525
GO:0042981	Regulation of apoptotic process	0.318025216	0.001956947

**Table 3:** PCC and GS of signature trophic receptors.

Receptor	moduleColor	PCC.cellcycle	GS.AD	p.GS.AD
GABRG2	Turquoise	0.419343809	-0.4121841	4.61621E-14
PGRMC1	Turquoise	0.405662742	-0.3663381	3.24182E-11
EPHA4	Turquoise	0.404438747	-0.3652521	3.74087E-11
MAGED1	Turquoise	0.404022816	-0.3860747	2.18463E-12
GRIA2	Turquoise	0.394280106	-0.3667586	3.06654E-11
CHRM1	Turquoise	0.381313001	-0.3271977	4.06247E-09
TNFRSF1B	Turquoise	0.353972271	0.3201443	9.04973E-09
GRIN2A	Turquoise	0.352962267	-0.4108274	5.68839E-14
TNFRSF1A	Turquoise	0.341193899	0.3440933	5.47841E-10
RXRA	Turquoise	0.339039633	0.3940831	6.93657E-13

AD: Alzheimer's disease; GS: gene significance; PCC: Pearson correlation coefficient.

## Data Availability

The data (including GSE132903, GSE118553, GSE5281, GSE37264, and GSE36980) generated and/or analyzed in this study are openly available in GEO (https://www.ncbi.nlm.nih.gov/geo/).

## Supplementary Materials

Supplementary 1 Supplementary Table S1: clinical phenotypic data of samples.

Supplementary 2 Supplementary Table S2: list of cell cycle genes.

Supplementary 3 Supplementary Table S3: list of trophic factor receptors.

Supplementary 4 Supplementary Table S4: differential expression of cell cycle-related genes.

Supplementary 5 Supplementary Table S5: PCC between cell cycle genes and trophic receptors. PCC: Pearson correlation coefficient.
